# Oral Administration of Zinc Sulfate with Intramuscular Foot-and-Mouth Disease Vaccine Enhances Mucosal and Systemic Immunity

**DOI:** 10.3390/vaccines12111268

**Published:** 2024-11-09

**Authors:** Min Ja Lee, Seokwon Shin, Hyeong Won Kim, Mi-Kyeong Ko, So Hui Park, Su-Mi Kim, Jong-Hyeon Park

**Affiliations:** Center for Foot-and-Mouth Disease Vaccine Research, Animal and Plant Quarantine Agency, 177 Hyeoksin 8-ro, Gimcheon-si 39660, Gyeongsangbuk-do, Republic of Korea; seokwon1218@korea.kr (S.S.); khw6848@korea.kr (H.W.K.); mkk80@korea.kr (M.-K.K.); sohui33@korea.kr (S.H.P.); belief@korea.kr (S.-M.K.); parkjhvet@korea.kr (J.-H.P.)

**Keywords:** mucosal immunity, systemic immunity, secretory IgA, zinc sulfate, foot-and-mouth disease

## Abstract

**Background/Objectives**: Foot-and-mouth disease (FMD) remains a significant global threat to livestock farming. Current commercial FMD vaccines present several challenges, including the risk of infection and adverse injection site reactions due to oil-based adjuvants. The complex immune environment of the gut-associated lymphoid tissue has the potential to induce broad and diverse immune responses. Therefore, we aimed to explore the potential of zinc sulfate as an oral adjuvant to enhance intestinal mucosal immunity and complement the effects of intramuscular (IM) FMD vaccination. **Methods**: We conducted serological analyses on mice and pigs, measuring secretory IgA (sIgA) levels and evaluating the expression of mucosal immunity-related genes in pigs. These assessments were used to investigate the systemic and mucosal immune responses induced by oral zinc sulfate administration in combination with an IM FMD vaccine. **Results**: This combination strategy significantly increased structural protein antibody titers and virus neutralization titers in experimental animals (mice) and target animals (pigs) across early, mid-, and long-term periods. Additionally, this approach enhanced the expression of key cytokines associated with mucosal immunity and increased sIgA levels, which are critical markers of mucosal immunity. **Conclusions**: Oral zinc sulfate administration may synergize with inactivated FMD vaccines, leading to sustained and enhanced long-term immune responses. This novel strategy could reduce the frequency of required vaccinations or allow for a lower antigen dose in vaccines, effectively stimulating the mucosal immune system and boosting systemic immunity. This approach has the potential to improve the overall efficacy of commercial FMD vaccines.

## 1. Introduction

Foot-and-mouth disease (FMD) remains a significant global threat to livestock farming despite extensive research on the disease’s characteristics, viral properties, and vaccine development since its discovery. Considerable costs are incurred in ongoing surveillance, vaccine development, and quality control of animals susceptible to FMD [1]. An ideal vaccine should be safe for the host, cost-effective, and capable of inducing protective immunity with a single dose. However, existing FMD vaccines use inactivated viruses as antigens and do not provide substantial protection until antibodies are produced following vaccination. In pigs, antibody titers are relatively lower than in cattle, with high inter-individual variation. Additionally, the oil-based adjuvant included in the vaccine causes side effects such as granulomas and fibrosis at the injection site, limiting the vaccine’s effectiveness against FMD [2].

Vaccines can be administered through routes such as intramuscular (IM), subcutaneous (SC), and intradermal (ID) injection. Ideally, vaccinations should be administered at the pathogen’s entry site to induce an effective host defense. Many pathogens invade the host through mucosal surfaces, making vaccine delivery a critical factor for successful immunization. Therefore, effective stimulation of mucosal immunity through vaccines or alternative means is an essential goal in FMD defense mechanisms [3]. The mucosal route of administration is more effective than injection routes in protecting against pathogens transmitted via mucosal surfaces [4]. However, limited mucosal vaccines are currently available on the market due to challenges such as antigen degradation in the extreme mucosal environment, individual differences in immune status, a lack of effective mucosal adjuvants, and unpredictable absorption [5,6,7].

Common pathways for stimulating mucosal immunity include the oral cavity, nasal cavity, rectum, vagina, and sublingual region, each representing a complex immune network across different mucosal surfaces [8]. Nasal mucosal stimulation induces a robust, systemic immune response via the nasal-associated lymphoid tissue (NALT) in the oral cavity, nasal cavity, and respiratory tract; however, NALT stimulation does not stimulate gut-associated lymphoid tissue (GALT) [9]. Although the intranasal (IN) route can elicit a stronger immune response at a lower dose than that induced by the oral route, side effects such as central nervous system paralysis and facial paralysis may occur. Therefore, further studies are needed to assess the accuracy and safety of IN administration. Vaginal-associated lymphoid tissue (VALT) stimulation can induce an effective local secretory IgA (sIgA) response; however, this remains understudied [10,11]. The oral route is considered ideal for stimulating GALT due to its ease of application in animals [12]. This route induces an immune response in the Peyer’s patches (PPs), leading to significant systemic sIgA production in the small intestine, colon, and saliva [13]. However, oral vaccination requires a larger quantity of antigens compared to other routes, and obstacles such as digestive enzymes and well-developed immune tolerance must be overcome. These issues make it difficult to induce a strong immune response and present significant barriers to vaccine development using inactivated FMD antigens [12]. Despite these limitations, the intestinal mucosa acts as a barrier to prevent harmful substances from entering the digestive system while also serving as a selective filter, diverting necessary dietary nutrients, water, and electrolytes from the lumen to intestinal blood circulation. Furthermore, the intestinal mucosa recognizes harmless food antigens as foreign substances but selectively filters them into circulation without triggering a protective immune response in the intestine. Thus, the intestinal mucosa can induce immune tolerance, making it an attractive target to stimulate GALT and induce an immune response, thereby enhancing the host immune system [14].

Zinc is an essential trace element that is vital for various physiological processes and for the development of the cell-mediated innate immune system. It functions as an antioxidant and anti-inflammatory molecule, making it crucial for promoting health [15,16]. Zinc is commonly added to both plant and animal supplements and is important in agriculture, medicine, and industry. Zinc deficiency can hinder optimal growth during pregnancy and in young animals. Zinc sulfate is generally used to address zinc deficiency [17,18], as it exhibits low toxicity in humans and causes no adverse effects in mice at doses up to 458 mg/kg/day. Additionally, zinc sulfate aids in repairing damaged intestinal cells. Furthermore, zinc sulfate can be effectively administered via various routes, including oral mucosal delivery [19,20]. Therefore, we aimed to evaluate the potential of zinc sulfate as an oral immunoadjuvant to stimulate intestinal mucosal immunity and complement IM FMD vaccination.

Specifically, we combined IM FMD vaccination with the oral administration of a zinc sulfate solution. We investigated humoral immune responses and assessed the efficacy of zinc sulfate in stimulating intestinal mucosal immunity through sIgA expression. This vaccination program was evaluated for its potential to address the limitations of commercial FMD vaccines.

## 2. Materials and Methods

### 2.1. Zinc Sulfate

Zinc sulfate was purchased from Sigma-Aldrich (St. Louis, MO, USA).

### 2.2. Cells and Antigen Purification

Baby hamster kidney (BHK-21) cells and fetal porcine kidney (LF-BK) cells were grown in Dulbecco’s Modified Eagle’s Medium (DMEM; HyClone, Logan, UT, USA), while fetal goat tongue epithelium (ZZ-R) cells were maintained in DMFM-F12 (HyClone). Both media were supplemented with 5% fetal bovine serum and 1% antibiotic–antimycotic solution (Gibco, Grand Island, NY, USA). The cell cultures were incubated at 37 °C in a humidified environment with 5% CO_2_.

Antigen purification was conducted following a modified protocol based on the method outlined by Lee et al. [21]. Briefly, FMDV O PA2 and A YC viruses were prepared by infecting BHK-21 cells in DMEM (HyClone). The obtained viruses were inactivated through two reactions with 0.003 N binary ethyleneimine (Sigma-Aldrich), each lasting 24 h. The inactivated viruses were then precipitated using polyethylene glycol 6000 (Sigma-Aldrich). The 146S antigen was isolated via a sucrose density gradient of 15–45%, followed by ultracentrifugation at 30,000 rpm for 4 h at 4 °C (SW 41Ti, Beckman Coulter, Fullerton, CA, USA). An inactivation test conducted with ZZ-R and BHK-21 cells verified that no live viruses remained in the inactivated supernatants.

### 2.3. Composition of Test Vaccine

Each dose of the test vaccine administered to mice included 0.375 μg of each FMDV O PA2 and A YC antigen (1/40 of the pig dose, 15 μg/dose/mL/pig). Additionally, the vaccine included ISA 206 [50% (*w*/*w*); Seppic, Paris, France], 10% aluminum hydroxide [Al(OH)_3_], and 15 μg Quil-A (InvivoGen, San Diego, CA, USA). The total volume of the vaccine administered to each mouse was 100 μL.

Each dose of the test vaccine administered to pigs contained 15 μg of each FMDV O PA2 and A YC antigen. The pig vaccine also included ISA 206 [50% (*w*/*w*); Seppic], 10% Al(OH)_3_, and 150 μg Quil-A (InvivoGen). The total volume of the vaccine administered to each pig was 1 mL.

### 2.4. Animals, Vaccination, and Oral Zinc Sulfate Administration

Naïve mice (C57BL/6, female, 6–7 weeks old) were obtained from KOSA BIO Inc. (Namyangju-si, Republic of Korea). Landrace pigs (8–9 weeks old), seronegative for antibodies against FMDV types O and A, were supplied by BARON BIO Inc. (Chilgok-gun, Republic of Korea). All animal care was conducted following previously established protocols [21]. Throughout the study, all animals were maintained in a specific pathogen-free biosafety level 3 facility dedicated to the Animal and Plant Quarantine Agency (APQA) and were allowed a minimum adaptation period of one week prior to the start of the study.

A total of five animals per group (mice and pigs) were randomly divided into three distinct groups. The negative control (NC) group received phosphate-buffered saline (PBS) treatment. The positive control (PC) group was administered the test vaccine via IM injection. The experimental (Exp.) group received oral zinc sulfate following the IM vaccination with the test vaccines. Mice were intramuscularly injected in the thigh with 100 µL of the test vaccine (PC and Exp.) or PBS (NC). Pigs were immunized intramuscularly twice, into the deep neck muscles with a 28-day interval, receiving 1 mL of the test vaccine (PC or Exp.) or PBS (NC).

All animals received daily oral administrations until 28 dpv and then at weekly intervals from 28 to 56 dpv. Mice and pigs in the Exp. group were orally administered 100 µg/100 µL and 20 mg/4 mL zinc sulfate solutions, respectively. The NC and PC groups received equivalent volumes of PBS instead of the zinc sulfate solution.

### 2.5. Food Efficiency Ratio and Systemic and Mucosal Immune Responses in Mice

The food efficiency ratio (FER) in mice was measured based on their food intake and body weight. For 56 days post-vaccination (dpv), food consumption and body weight were monitored weekly. The FER was calculated using the following formula:

$$FER=\frac{Weight\;gain\;(grams)}{Food\;intake\;(grams)}\times 100$$

To evaluate the impact of oral zinc sulfate on adaptive and mucosal immune responses in mice, blood samples were collected at various time points throughout 84 dpv, specifically on days 0, 7, 14, 21, 28, 35, 42, 56, 70, and 84. These samples were examined for antibodies targeting structural proteins (SPs) O and A, virus neutralization (VN) titers, and sIgA levels. All serum samples were stored at −80 °C until they were ready for analysis.

### 2.6. FMDV Challenge After Oral Zinc Sulfate Administration in Mice

Challenge experiments were conducted to verify the protective efficacy of combined oral zinc sulfate and IM vaccination against FMD. The mice were vaccinated intramuscularly at 0 dpv. Zinc sulfate was administered orally in conjunction with the vaccination. At 84 dpv, the mice were challenged with FMDV (100 LD_50_ of O/VET/2013) via intraperitoneal injection. Survival rates and changes in body weight were monitored in the experimental animals for up to 7 days post-challenge (dpc). Body weight was measured daily at the same time and under consistent conditions for 7 days. Each individual animal’s body weight at the time of the viral challenge (0 dpc) was established as the baseline at 100%. After measuring the individual body weight (g), the values were converted to percentages to monitor each animal’s weight changes. Animals that experienced a weight loss of more than 20% compared to the baseline (100%) at 0 dpc were considered deceased and euthanized in accordance with our institution’s IACUC guidelines, with due consideration for animal welfare. Mice were euthanized using carbon dioxide (CO_2_) inhalation, with the CO_2_ flow replacing 30% to 70% of the cage volume per minute. Euthanasia was performed under deep anesthesia through extensive intracardiac perfusion with cold potassium chloride (2 mM KCl/kg) to prevent the risk of reawakening after CO_2_ anesthesia.

### 2.7. Biochemical Assays and Systemic and Mucosal Immune Response in Pigs

The following serum biochemical parameters were analyzed: alanine aminotransferase (ALT), aspartate aminotransferase (AST), blood urea nitrogen (BUN), creatinine (CREA), lactate dehydrogenase (LDH), total protein (TP), albumin (ALB), and the albumin-globulin (A/G) ratio. These analyses were conducted using a HITACHI Automatic Analyzer 3100 (Hitachi High-Tech Corporation, Tokyo, Japan), provided by KLS Bio-Inc. (Uijeongbu, Republic of Korea).

To evaluate the effect of oral zinc sulfate on adaptive and mucosal immune responses in pigs, blood samples were collected at 0, 7, 14, 21, 28, 35, 42, 56, 70, and 84 dpv, and all analyses were performed as described in Section 2.5.

### 2.8. Ethics Statement

Approval for the study and related experimental protocols was granted by the APQA Ethics Committee under the accreditation numbers IACUC 2023-719 and 2024-863.

### 2.9. Serological Assays

All serological assays were performed by previously established protocols [21]. To evaluate the serum antibody levels of SP O and A, Enzyme-Linked Immunosorbent Assay (ELISA) kits designed for FMDV types O and A (PrioCheck™, Prionics AG, Schlieren, Switzerland) were utilized with undiluted serum in accordance with the manufacturer’s guidelines. Absorbance readings were taken at 450 nm using a spectrophotometer (Hidex, Turku, Finland), and the results were expressed as percent inhibition (PI) values. Animals were classified as seropositive if the PI value surpassed 50%, based on the criteria provided by the PrioCheck™ FMDV kit.

The VN test was performed following the protocols established by the World Organisation for Animal Health (WOAH). Serum samples from the vaccinated mice were inactivated by heating at 56 °C for 30 min. The sera were then diluted in a two-fold series (from 1:8 to 1:1024) and subsequently exposed to FMDV type O (O PA2) or FMDV type A (A YC) at a concentration of 100 TCID_50_/0.5 mL, followed by incubation for 1 h at 37 °C. LF-BK cells were added to all the wells, and the cytopathic effects were observed after 72 h. VN titers were expressed as Log_10_ of the reciprocal of the serum dilution required to neutralize 100 TCID_50_ of the virus.

Murine and porcine serum sIgA levels were quantified using an ELISA kit (Cusabio^®^, Wuhan, China) following the manufacturer’s instructions. Absorbance readings were obtained at 450 nm with a spectrophotometer (Hidex).

### 2.10. Gene Evaluation of Porcine Blood Specimens for Mechanistic Assessments

To elucidate the mechanisms underlying the immune responses triggered by stimulation of the intestinal mucosa via the oral administration of zinc sulfate, we conducted experiments in line with previously established protocols [21]. Peripheral blood mononuclear cells (PBMCs) were isolated from porcine blood samples collected at 0 and 14 dpv from pigs (*n* = 5/group) using the procedures described in Section 2.4. Isolation was carried out with heparin-coated tubes (Becton Dickinson Company, Franklin Lakes, NJ, USA) and Lymphoprep tubes (Stem Cell Technologies, Vancouver, BC, Canada). RNA extraction from the purified porcine PBMCs was performed using an RNeasy Mini Kit (QIAGEN, Hilden, Germany) in conjunction with TRIzol reagent (Invitrogen, Middlesex County, MA, USA). cDNA synthesis was achieved utilizing the GoScript Reverse Transcription System (Promega, Madison, WI, USA), following the manufacturer’s guidelines. Amplification was executed on a CFX96™ Touch quantitative real-time polymerase chain reaction (qRT-PCR) system (Bio-Rad, Hercules, CA, USA) using SYBR Green Supermix (Bio-Rad). Gene quantification was normalized to *HPRT* (an endogenous housekeeping gene) and expressed relative to control values. Detailed information on the primers used can be found in Supplementary Table S1.

### 2.11. Statistical Analyses

Quantitative data are expressed as the mean ± standard error of the mean (SEM), unless indicated otherwise. Group differences were evaluated using either one-way or two-way analysis of variance, followed by Tukey’s or Dunnett’s post hoc test. Statistical significance was indicated as * *p* < 0.05, ** *p* < 0.01, *** *p* < 0.001, and **** *p* < 0.0001. Parametric tests were employed for group comparisons, while survival curves were constructed using the Kaplan–Meier method, with differences assessed via the log-rank test. All statistical analyses were conducted using Prism version 10.0.2 (GraphPad, San Diego, CA, USA).

## 3. Results

### 3.1. Effect of Oral Zinc Sulfate Administration on Body Weight, Food Intake, and FER

To evaluate the effect of oral zinc sulfate administration on FER in experimental animals (mice), changes in body weight were measured (Supplementary Table S2). The FER of the NC group was the highest; however, there was no significant difference among the three groups (Supplementary Table S3).

### 3.2. Oral Zinc Sulfate Administration Induces and Sustains Systemic Immune Responses in Mice and Protects against FMDV

**Figure 1:**
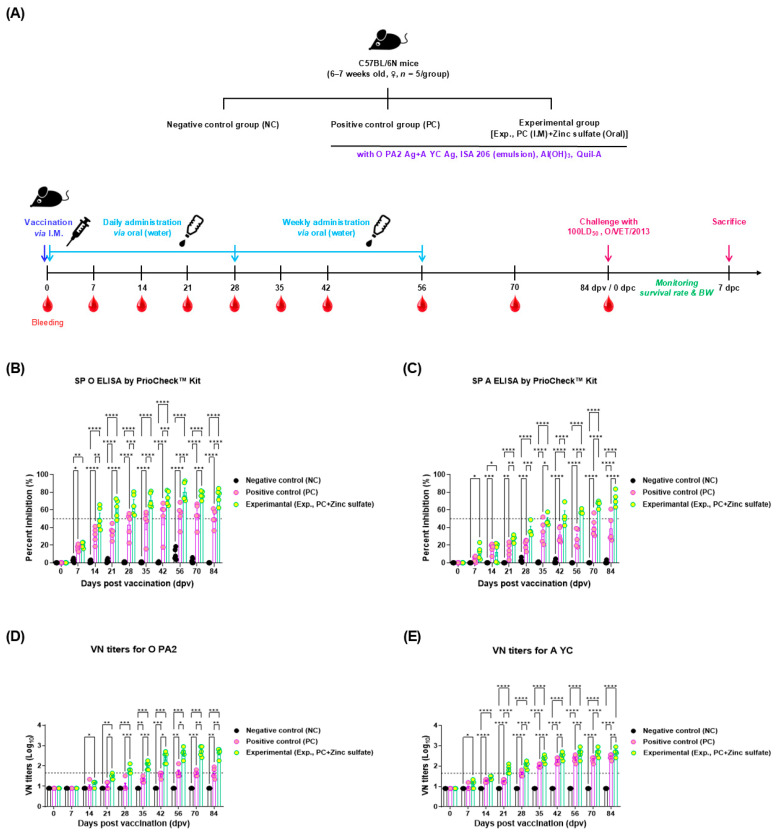
Oral zinc sulfate administration influences early, mid-, and long-term immune responses in mice. (**A**–**E**) Overview of experimental strategy (**A**); antibody titers for SP O measured using PrioCheck™ FMDV kit with undiluted serum (**B**); antibody titers for SP A assessed using PrioCheck™ FMDV kit with undiluted serum (**C**); VN titers for O PA2 (**D**); and VN titers for A YC (**E**). The dotted line on the y-axis in (**B**,**C**) indicates the positive threshold provided by the manufacturer. The dotted line on the y-axis in (**D**,**E**) indicates the 1:45 (1.65 Log_10_) virus-neutralizing titer cutoff level recognized as the defensive level against virus infection. Data are expressed as the mean ± standard error of the mean (SEM) based on triplicate measurements (*n* = 5/group). Statistical evaluations were conducted using two-way analysis of variance (ANOVA), followed by Tukey’s post hoc test. Significance levels are indicated as * *p* < 0.05, ** *p* < 0.01, *** *p* < 0.001, and **** *p* < 0.0001.

To evaluate the effect of oral zinc sulfate administration on the long-term humoral immune response in experimental animals, an experiment was conducted according to the design shown in Figure 1A. The antigen-specific antibody titers measured in undiluted serum using the SP O ELISA were significantly higher in the Exp. group than in the PC group, from 14 dpv for SP O and from 21 dpv for SP A to 84 dpv (Figure 1B,C). The VN titers for O PA2 or A YC were significantly higher in the Exp. group than in the PC group, from 21 to 84 dpv (Figure 1D,E).

**Figure 2:**
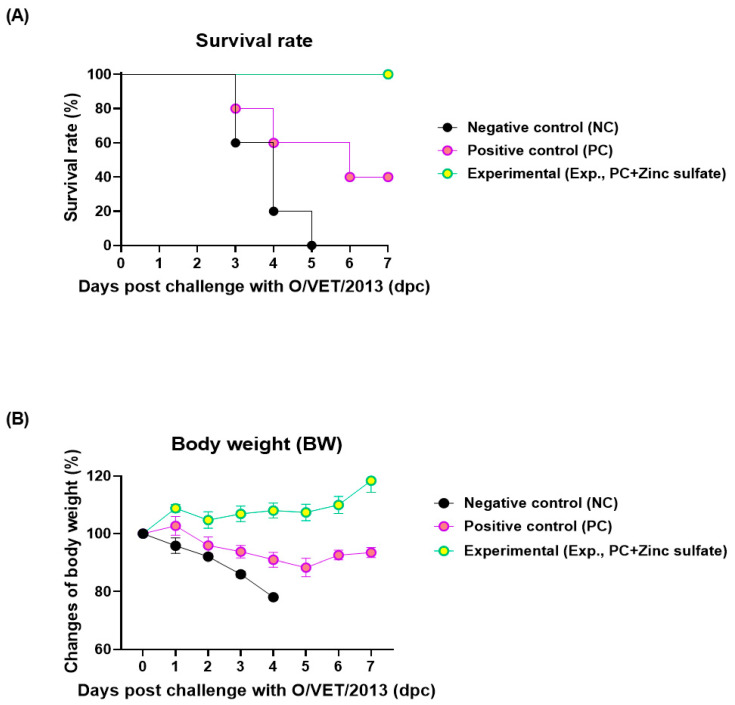
Oral administration of zinc sulfate improves host protection against virus in FMD-vaccinated mice. (**A**,**B**) Survival rates post-challenge with O/VET/2013 (**A**); Changes in body weight post-challenge with O/VET/2013 (**B**). Data are expressed as mean ± standard error of mean (SEM) based on triplicate measurements (*n* = 5/group).

The protective effects of the FMD vaccine combined with oral zinc sulfate against viral infections were also assessed (Figure 1A). The Exp., PC, and NC groups showed survival rates of 100%, 40%, and 0%, respectively (Figure 2A). Furthermore, all mice in the NC group lost more than 20% body weight and died by 5 dpc. In contrast, three mice died in the PC group by 6 dpc, and the surviving mice in the PC group lost about 10% body weight by 6 dpc but regained it afterwards. The Exp. group did not show any weight loss (Figure 2B).

### 3.3. Oral Zinc Sulfate Administration Induces and Sustains a Systemic Immune Response in Pigs

Serum biochemical tests were conducted to assess the potential toxicity of orally administered zinc sulfate. Except for AST levels, the levels of all other parameters (ALT, BUN, CREA, LDH, TP, ALB, and A/G ratio) did not differ significantly among the three groups. At 84 dpv, AST levels in the Exp. group were significantly lower than those in the NC group (Table 1).

**Figure 3:**
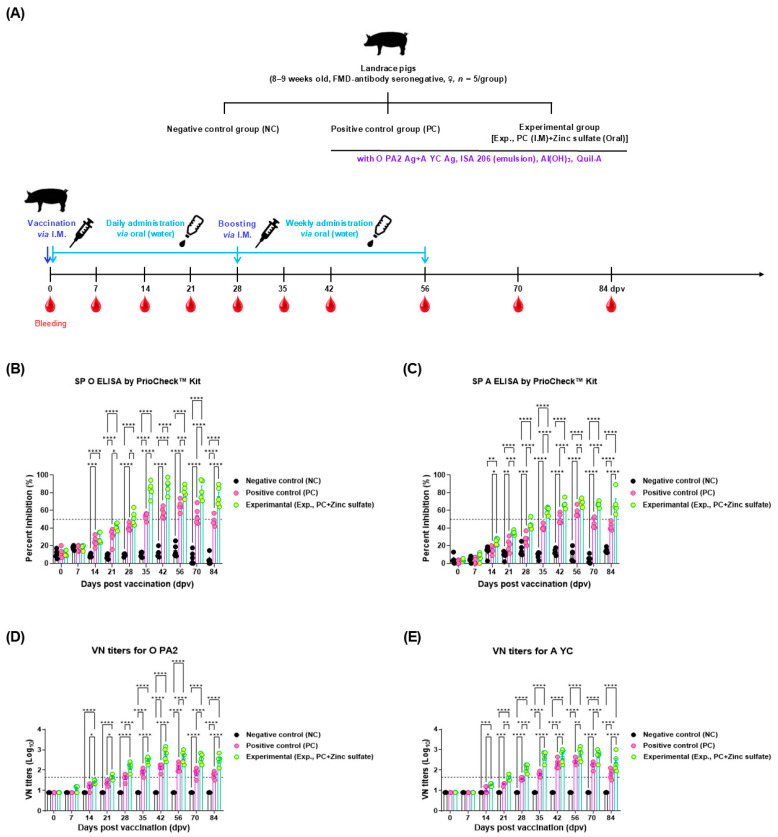
Oral zinc sulfate administration influences early, mid-term, and long-term immune responses in pigs. (**A**–**E**) An overview of the experimental strategy (**A**); antibody titers for SP O measured using the PrioCheck™ FMDV kit (**B**); antibody titers for SP A assessed with the PrioCheck™ FMDV kit (**C**); VN titers for O PA2 (**D**); and VN titers for A YC (**E**). The dotted line on the y-axis in (**B**,**C**) indicates the positive threshold provided by the manufacturer. The dotted line on the y-axis in (**D**,**E**) indicates the 1:45 (1.65 Log_10_) virus-neutralizing titer cutoff level recognized as the defensive level against virus infection. Data are expressed as the mean ± standard error of the mean (SEM) based on triplicate measurements (*n* = 5/group). Statistical evaluations were conducted using two-way analysis of variance (ANOVA), followed by Tukey’s post hoc test. Significance levels are indicated as * *p* < 0.05; ** *p* < 0.01; *** *p* < 0.001; and **** *p* < 0.0001.

The effect of oral zinc sulfate administration on long-term humoral immune responses was evaluated in the study animals (Figure 3A). The antigen-specific antibody titers measured using the SP O and A ELISA and VN tests were significantly higher in the Exp. group than in the PC group, from 14 dpv or 21 dpv to 84 dpv (Figure 3B–E). Notably, the antibody and VN titers decreased in the PC group after 56 dpv, whereas those in the Exp. group remained at a high level.

### 3.4. Oral Zinc Sulfate Administration Enhances Mucosal Immune Responses in Mice and Pigs

**Figure 4:**
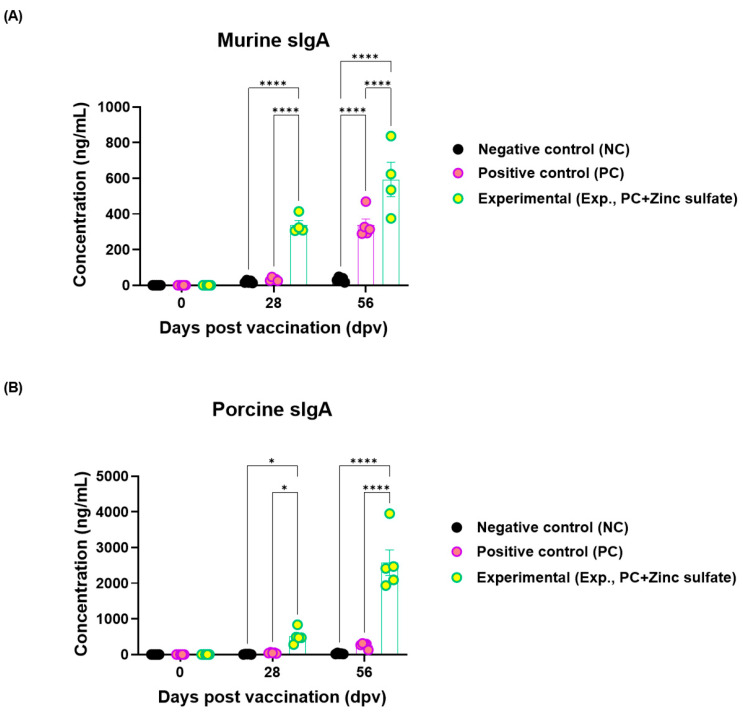
Oral zinc sulfate administration enhances the expression of secretory IgA. (**A**,**B**) sIgA antibody levels in mice (Cusabio^®^ mouse secretory IgA ELISA kit) (**A**); sIgA antibody levels in pigs (Cusabio^®^ pig secretory IgA ELISA kit) (**B**). Data are expressed as the mean ± standard error of the mean (SEM) based on triplicate measurements (*n* = 5/group). Statistical evaluations were conducted using two-way analysis of variance (ANOVA), followed by Tukey’s post hoc test. Significance levels are indicated as * *p* < 0.05; and **** *p* < 0.0001.

An sIgA ELISA was performed to evaluate the effect of oral zinc sulfate administration on sIgA expression in mice and pigs. In both mice and pigs, the Exp. group showed significantly increased sIgA levels compared to those in the NC and PC groups at 28 and 56 dpv (Figure 4A,B).

### 3.5. Oral Zinc Sulfate Administration Stimulates Mucosal Immune Responses by Upregulating Mucosal Immunity-Related Cytokines in PBMCs

**Figure 5:**
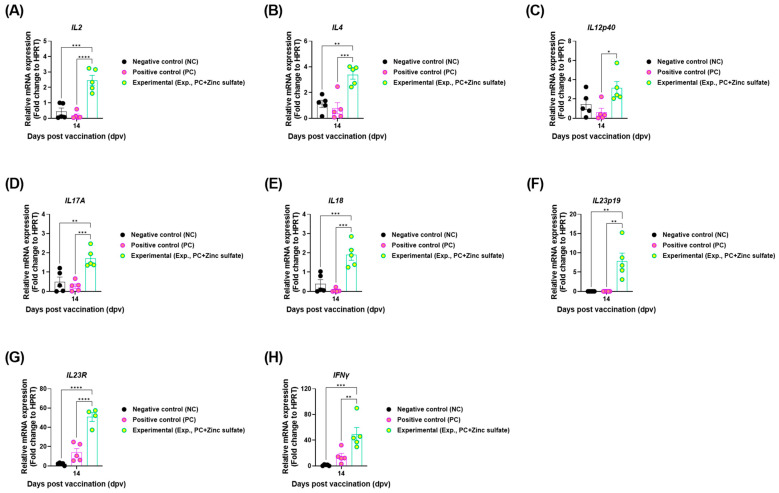
Oral zinc sulfate administration boosts the expression of mucosal immune-related cytokines in pigs. (**A**–**H**) Gene expression levels of *IL*2 (**A**); *IL4* (**B**); *IL12p40* (**C**); *IL17A* (**D**); *IL18* (**E**); *IL23p19* (**F**); *IL23R* (**G**); *IFNγ* (**H**). Data are expressed as the mean ± standard error of the mean (SEM) based on triplicate measurements (*n* = 5/group). Statistical evaluations were conducted using two-way analysis of variance (ANOVA), followed by Tukey’s post hoc test. Significance levels are indicated as * *p* < 0.05; ** *p* < 0.01; *** *p* < 0.001; and **** *p* < 0.0001.

To investigate the underlying mechanisms of mucosal immune responses induced by oral zinc sulfate administration, qRT-PCR was performed using porcine PBMCs (Figure 3A). The expression levels of cytokines, including interleukin (*IL)2*, *IL4*, *IL12p40*, *IL17A*, *IL18*, *IL23p19*, *IL23R*, and *IFNγ* were significantly higher in the Exp. group than in the PC and NC groups (Figure 5A–H). No significant differences were observed between the PC and NC groups.

## 4. Discussion

FMD is a serious veterinary infectious disease, even in countries with advanced livestock farming systems. Despite the use of inactivated FMD antigens in vaccines, the disease remains globally prevalent. Efforts are underway to enhance the safety and efficacy of these vaccines due to various side effects, such as non-persistent antibody titers and local reactions at the injection site, that may occur following FMD vaccination [22,23]. While FMD vaccines can reduce disease severity and viral shedding, asymptomatic hosts can still spread the virus. Additionally, the production and distribution of vaccines for large-scale vaccination programs are very expensive [24,25]. Therefore, there is an urgent need for effective and innovative strategies to control FMD. The oral administration of harmless immunostimulants can stimulate the intestinal mucosa and activate the mucosal immune system, thus contributing to systemic immunity [26]. Hence, we focused on the oral administration of zinc sulfate.

Zinc is an essential trace nutrient crucial for numerous biological processes, including DNA replication, signal transduction, and cell proliferation. Zinc ions play a vital role in both innate and adaptive immunity [27,28,29,30]. Zinc helps induce T cell activation through non-covalent interactions with CD4^+^ and CD8^+^ T cells, and its deficiency can reduce the function and production of Th and cytotoxic T cells [31,32]. We evaluated the efficacy of zinc sulfate as an adjuvant by administering a vaccine formulation containing it via IM injection prior to oral administration. The inclusion of zinc sulfate as a vaccine adjuvant stimulated immune cells through pathogen recognition receptor activation, resulting in a robust systemic immune response, including the production of serum IgG and IgA. It also promoted the generation of pro-inflammatory cells and enhanced the production of cytokines associated with systemic and mucosal immunity [33].

The intestine is the largest immune organ in mammals and plays a critical role in interacting with dietary antigens, commensal microbes, and external pathogens [34]. Therefore, inducing immune responses through dietary interventions is advantageous. Additionally, the intestinal epithelial cells and mucosal layer provide a physical barrier to pathogen invasion. Moreover, the mucosal layer contains a higher concentration of dispersed lymphocytes, antigen-presenting cells (APCs), and more antibody-producing cells than those present in the spleen and lymph nodes combined [35,36]. We hypothesized that efficient stimulation of the intestine would improve overall host health by activating various immune responses and enhance the efficacy of FMD vaccines by modulating mucosal and systemic immune responses. Thus, we stimulated intestinal mucosal immunity by orally administering zinc sulfate to enhance the efficacy of FMD vaccines.

No significant differences in the FER measured in the experimental animals suggested that oral zinc sulfate administration did not affect body weight gain or feed intake, indicating its potential use in feed (Supplementary Tables S2 and S3). Additionally, oral zinc sulfate rapidly accumulated and increased the SP O, A antibody titers, and VN antibody titers in the experimental group, which were sustained over an extended period. It also positively impacted viral inactivation (Figure 1 and Figure 2). This trend was similarly observed in target animals (Figure 3). SP antibodies and VN titers are important indicators of the host’s adaptive immune defense. According to the guidelines for verifying FMD vaccine efficacy, a VN titer greater than 1.65 (Log_10_) indicates host protection from FMDV [21]. Serum biochemical analysis confirmed that zinc sulfate did not exhibit toxicity in the target animals and did not negatively affect liver and kidney function. Notably, the group receiving oral zinc sulfate exhibited low AST levels. An increase in blood concentration of AST, which is released into the bloodstream when the liver or muscle is damaged, serves as an important indicator of liver toxicity. The normal AST level for the Landrace pigs used in our experiments has been reported to be between 27.2 and 89.9 U/L [37,38]. The low AST levels indicated that oral zinc sulfate administration prevented liver damage.

The A/G ratio is used to evaluate liver function, nutritional status, and immune health. Globulin is calculated by subtracting albumin from the total protein [39]. These results suggest that oral zinc sulfate administration does not negatively affect the host; rather, it triggers a robust antibody-mediated (humoral) immune response and can establish long-term immunity in target animals.

Subsequently, we evaluated the expression levels of murine and porcine sIgA using ELISA to determine whether oral zinc sulfate administration stimulates the intestinal mucosa and activates the mucosal immune system, thereby enhancing host defense. In the experimental and target animals, the group administered oral zinc sulfate (Exp. group) exhibited a marked increase in sIgA concentrations at 28 and 56 dpv (Figure 4). These findings suggest that oral zinc sulfate administration may stimulate the intestinal mucosal immunity of target animals, and the mucosal immune response could eventually trigger a systemic immune response, thereby enhancing the overall host immune response.

However, all experiments and analyses were conducted using serum, and no analyses were performed with mucosal specimens. The only evidence for mucosal immunity in this study was the sIgA levels measured in serum samples using an ELISA kit. In future studies, we plan to evaluate zinc sulfate as a feed additive and an oral vaccine adjuvant. We will also examine IgA secretion and its types across various mucosal tissues.

According to the Korean FMD vaccine evaluation criteria, a VN titer of 1.65 Log_10_ or higher indicates a protective effect against virus challenge [40]. The experimental group showed VN titers higher than 1.65 Log_10_ at 28 dpv, while the other groups did not (Figure 3), suggesting that oral zinc sulfate administration may ultimately contribute to the protection of the host against FMDV infection. GALT, a key component in gut mucosal immune reactions, consists of structures such as the appendix, PPs, and isolated lymphoid follicles [41]. The surface of PPs is lined with a specialized epithelial layer called the follicle-associated epithelium, which includes microfold (M) cells and forms a pocket structure containing numerous immune cells distinct from other epithelial cells [42]. When antigens are recognized through the uptake function of M cells, APCs such as dendritic cells (DCs) and macrophages (MΦs), which are abundant in the pocket, interact to induce the production of effector and memory B and T cells. Subsequently, antigen-specific B and T cells migrate through the lymphatic system and bloodstream to mucosal effector sites, where they interact to produce sIgA in cooperation with IgA-committed B cells, Th1 cells, Th2 cells, and regulatory T cells [6]. The sIgA produced serves as a crucial frontline defense against pathogens in mucosal immunity by preventing their adherence to the mucosa—a process known as immune exclusion—making it a key indicator of mucosal immunity [43].

To explore the mechanisms involved in enhancing both adaptive and mucosal immunity, we evaluated the gene expression levels of key mucosal immunity-related cytokines (*IL2*, *IL4*, *IL12p40*, *IL17A*, *IL18*, *IL23p19*, *IL23R*, and *IFNγ*), which are known to induce innate and adaptive immunity (Figure 5). M cells in the GALT uptake antigens and interact with DCs and MΦs in the pocket structure to promote the expression of IL4 and IL12 in naive T cells. Additionally, IL12p40 secreted by DCs induces IL12 secretion, contributing to T cell-mediated immunity. IL12 promotes the Th1 response, whereas IL4 promotes the Th2 response. IFNγ and IL2 produced by Th1 cells, along with IL4 and IL10 produced by Th2 cells, are crucial IgA-enhancing cytokines that preferentially activate sIgA^+^ B cells and lead to their final differentiation into IgA plasma cells [44,45,46]. IgA-committed B lymphocytes affected by these cytokines migrate through the mesenteric lymph nodes to mucosal sites, where they are involved in sIgA production [47]. Moreover, we detected increased expression of IL17A, which is an important cytokine involved in the generation of neutrophil extracellular traps (NETs). NETs consist of extracellular DNA and antimicrobial proteins that originate from the granules and nuclei of neutrophils and serve as key components of innate immunity by capturing various microorganisms [48]. We confirmed that oral zinc sulfate administration to target animals upregulated the expression of cytokines related to mucosal immunity, which could, in turn, activate the mucosal immune system.

In summary, we designed and evaluated a novel strategic FMD vaccine platform that combines oral zinc sulfate administration with IM FMD vaccination. This platform induced innate immune responses, increased and prolonged antibody and VN titers, stimulated the intestinal mucosa to enhance sIgA secretion, and upregulated the expression of mucosal immunity-related genes. This approach represents a promising innovation for FMDV prevention, as it may improve host health and prevent the spread of FMDV. Furthermore, this study confirms the potential use of zinc sulfate as an adjuvant for mucosal vaccines. Although the efficacy of oral zinc sulfate administration has been demonstrated using aqueous solutions, further studies are needed to integrate zinc sulfate into feed additives and evaluate its efficacy against diseases other than FMD, as well as its role as an adjuvant for mucosal vaccines.

In this study, two vaccinations, including a booster shot, were administered to the target animals. This approach was necessary because pigs exhibit greater inter-individual variation compared to experimental animals, such as mice, and other target animals, such as cattle, making it challenging to induce a consistent immune response. Based on these findings, future research should aim to reduce the amount of antigen or administer a single vaccine to assess the levels of antibodies and VN titers. Additionally, the focus will be on developing zinc sulfate-supplemented feed that can be conveniently used by farmers and evaluating its applicability in both experimental and target animals. Moreover, the ability of zinc sulfate to stimulate intestinal mucosal immunity and promote the production of sIgA suggests its potential as an adjuvant in oral vaccines. Leveraging these properties, zinc sulfate may exhibit significant efficacy in combating gastrointestinal diseases, such as porcine epidemic diarrhea and transmissible gastroenteritis virus, in target animals when added to oral vaccines. It may also have potential applications in bait vaccines.

## 5. Conclusions

A novel vaccine program that combines oral zinc sulfate administration with IM FMD vaccine administration consistently induced faster and more potent antibodies and VN titers due to the activation of B cells. Additionally, this strategy promoted the secretion of sIgA and mucosal immunity-related cytokines in the host. These results demonstrate that oral zinc sulfate administration positively influences the health status of the host and can induce both mucosal and systemic immune responses. Furthermore, these combination strategies could enhance the efficacy of commercial vaccines, reduce the antigen load in vaccines, and shorten the vaccination cycle. This program presents a new vaccine strategy that can be applied to various veterinary viral diseases, including FMD.

## Figures and Tables

**Table 1:** Biochemical assays in serum from pigs treated with oral zinc sulfate for 84 days post-vaccination. Data are represented by mean ± standard error of mean (SEM) of triplicate measurements (*n* = 5–6/group). Statistical analyses were performed using one-way ANOVA, followed by Tukey’s post hoc test. Different superscripts (^a,b^) represent significant differences at *p* < 0.05.

Group	Days Post-Vaccination (dpv)	ALT (U/L)	AST (U/L)	BUN (mg/dL)	CREA (mg/dL)	LDH (U/L)	TP (mg/dL)	ALB (mg/dL)	A/G ratio
NC	0	41.20 ± 3.68	40.00 ± 2.06	5.90 ± 0.37	0.75 ± 0.02	421.28 ± 45.74	2.58 ± 0.10	2.90 ± 0.06	1.22 ± 0.04
	28	56.80 ± 4.03	56.25 ± 22.48	9.56 ± 1.08	1.01 ± 0.05	496.23 ± 40.97	6.08 ± 0.15	3.36 ± 0.05	1.26 ± 0.08
	56	49.60 ± 1.85	33.40 ± 1.82	10.50 ± 1.21	1.15 ± 0.02	333.10 ± 13.02	6.72 ± 0.15	3.14 ± 0.08	0.89 ± 0.05
	84	47.00 ± 1.74	66.00 ± 14.04 ^a^	17.26 ± 1.47	1.46 ± 0.05	266.48 ± 2.76	6.06 ± 0.07	3.62 ± 0.04	1.49 ± 0.05
PC	0	37.20 ± 1.73	32.60 ± 0.61	7.50 ± 1.43	0.68 ± 0.02	340.30 ± 13.22	5.34 ± 0.07	2.98 ± 0.07	1.26 ± 0.08
	28	48.40 ± 1.85	45.50 ± 3.32	9.30 ± 0.67	0.93 ± 0.02	456.34 ± 17.62	6.30 ± 0.14	3.32 ± 0.11	1.12 ± 0.07
	56	53.80 ± 3.33	48.60 ± 9.20	13.98 ± 0.95	1.16 ± 0.05	361.90 ± 26.52	6.80 ± 0.10	3.36 ± 0.08	0.98 ± 0.02
	84	43.60 ± 2.79	37.75 ± 25.86	16.78 ± 1.60	1.30 ± 0.07	254.12 ± 8.38	6.34 ± 0.12	3.72 ± 0.09	1.44 ± 0.08
Exp.	0	42.40 ± 4.09	42.40 ± 1.95	4.92 ± 0.64	0.74 ± 0.03	310.00 ± 10.42	5.58 ± 0.10	2.84 ± 0.20	1.08 ± 0.13
	28	55.60 ± 5.12	39.40 ± 3.92	6.12 ± 0.96	1.03 ± 0.03	425.12 ± 23.09	6.42 ± 0.10	3.52 ± 0.15	1.26 ± 0.13
	56	57.60 ± 3.60	39.00 ± 3.97	11.38 ± 1.22	1.12 ± 0.02	327.82 ± 15.60	7.06 ± 0.45	3.24 ± 0.12	0.94 ± 0.14
	84	37.60 ± 2.41	32.00 ± 1.90 ^b^	14.70 ± 0.88	1.43 ± 0.09	251.16 ± 15.55	6.10 ± 0.34	3.34 ± 0.27	1.25 ± 0.15

dpv, days post-vaccination; NC, negative control; PC, positive control; Exp., experimental; ALT, alanine aminotransferase; AST, aspartate aminotransferase; BUN, blood urea nitrogen; CREA, creatinine; LDH, lactate dehydrogenase; TP, total protein; ALB, albumin; A/G ratio, albumin–globulin ratio.

## Data Availability

All data supporting the findings of this study are available from the corresponding author upon request.

## Supplementary Materials

The following supporting information can be downloaded at https://www.mdpi.com/article/10.3390/vaccines12111268/s1. Table S1: List of primer sequences for qRT-PCR. Table S2: Body weight changes in mice treated with zinc sulfate through oral administration at 56 days post-vaccination. Table S3: Weight gain and food efficiency ratio (FER) of mice treated with zinc sulfate by oral administration at 56 days post-vaccination.
