# Supplementary material for: Oral Administration of Zinc Sulfate with Intramuscular Foot-and-Mouth Disease Vaccine Enhances Mucosal and Systemic Immunity
Source: Vaccines (Basel). 2024 Nov 9;12(11):1268. doi: 10.3390/vaccines12111268 (PMC11598382; doi:10.3390/vaccines12111268)
Supplement: Supplementary file 1 [file vaccines-12-01268-s001.zip › vaccines-3275840-supplementary.pdf]

## Supplementary Table legend

**Table S1: List of primer sequences for qRT-PCR.**

**Table S2: Body weight changes in mice treated with zinc sulfate through oral administration at 56 days post-vaccination.**

Data are expressed as the mean ± standard error of the mean (SEM) based on triplicate measurements (*n* = 5/group). Statistical evaluations were conducted using two-way analysis of variance (ANOVA), followed by Tukey's post-hoc test.

dpv, days post vaccination; NC, negative control; PC, positive control; Exp., experimental

**Table S3: Weight gain and food efficiency ratio (FER) of mice treated with zinc sulfate by oral administration at 56 days post-vaccination.**

Data are expressed as the mean ± standard error of the mean (SEM) based on triplicate measurements (*n* = 5/group). Statistical evaluations were conducted using two-way analysis of variance (ANOVA), followed by Tukey's post-hoc test.

FER, food efficiency ratio; dpv, days post vaccination; NC, negative control; PC, positive control; Exp., experimental

**Table S1.**

| Target | Forward/Reverse | Sequence (5'-3') | Length (mer) |
|---|---|---|---|
| *IL2* | *IL2* F | AAGCTCTGGAGGGAGTGCTA | 20 |
| | *IL2* R | CAACAGCAGTTACTGTCTCATCA | 23 |
| *IL4* | *IL4* F | CTCACCTCCCAACTGATCCC | 20 |
| | *IL4* R | TGTGTCCGTGGACGAAGTTG | 20 |
| *IL12p40* | *IL12p40* F | GGAGTATAAGAAGTACAGAGTGG | 23 |
| | *IL12p40* R | GATGTCCCTGATGAAGAAGC | 20 |
| *IL17A* | *IL17A* F | CTCGTGAAGGCGGGAATCAT | 20 |
| | *IL17A* R | GGTGTGCTCCGGTTCAAGAT | 20 |
| *IL18* | *IL18* F | AGCTGAAAACGATGAAGACCTG | 22 |
| | *IL18* R | AAACACGGCTTGATGTCCCT | 20 |
| *IL23p19* | *IL23p19* F | CCATATCCAGTGCGGGGATG | 20 |
| | *IL23p19* R | AGGCCTTGGTGGATCCTTTG | 20 |
| *IL23R* | *IL23R* F | TCCCTCATTGCAAAGCACAA | 20 |
| | *IL23R* R | GCATCTCCTCTTGCAAGCAAAT | 22 |
| *IFNγ* | *IFNγ* F | GCCATTCAAAGGAGCATGGAT | 21 |
| | *IFNγ* R | CTGATGGCTTTGCGCTGGAT | 20 |
| *HPRT* | *HPRT* F | CCCAGCGTCGTGATTAGTGA | 20 |
| | *HPRT* R | GCCGTTCAGTCCTGTCCATA | 20 |

**Table S2.**

| Group | 0 dpv | 7 dpv | 14 dpv | 21 dpv | 28 dpv | 56 dpv |
|---|---|---|---|---|---|---|
| NC | 18.68±0.28 | 19.93±0.38 | 20.85±0.42 | 21.39±0.43 | 21.54±0.31 | 23.13±0.60 |
| PC | 19.10±0.34 | 20.84±0.31 | 21.00±0.49 | 21.57±0.45 | 21.70±0.46 | 24.01±0.36 |
| Exp. | 19.46±0.26 | 20.76±0.33 | 19.85±0.94 | 20.69±0.46 | 20.48±0.85 | 23.58±0.48 |

**Table S3.**

| Group | Weight gain (g/56dpv) | Food intake (g/56dpv) | FER |
|---|---|---|---|
| NC | 4.45±0.61 | 121.57 | 3.66±0.50 |
| PC | 4.85±0.58 | 152.86 | 3.17±0.38 |
| Exp. | 4.00±0.36 | 148.92 | 2.68±0.24 |
